# Effects of vitamin D deficiency on neurobehavioural outcomes in children: a systematic review

**DOI:** 10.12688/wellcomeopenres.15730.2

**Published:** 2020-06-11

**Authors:** Agnes M. Mutua, Reagan M. Mogire, Alison M. Elliott, Thomas N. Williams, Emily L. Webb, Amina Abubakar, Sarah H. Atkinson

**Affiliations:** 1 KEMRI-Wellcome Trust Research Programme, Kilifi, 80108, Kenya; 2 Department of Public Health, School of Human and Health Sciences, Pwani University, Kilifi, 80108, Kenya; 3 Open University, KEMRI-Wellcome Trust Research Programme - Accredited Research Centre, Kilifi, 80108, Kenya; 4 Medical Research Council / Uganda Virus Research Institute and London School of Hygiene and Tropical Medicine Uganda Research Unit, Entebbe, Uganda; 5 Department of Clinical Research, London School of Hygiene and Tropical Medicine, London, WC1E 7HT, UK; 6 Department of Medicine, Imperial College London, London, W21N, UK; 7 Centre for Tropical Medicine and Global Health, Nuffield Department of Medicine, University of Oxford, Oxford, OX3 7FZ, UK; 8 MRC Tropical Epidemiology Group, Department of Infectious Disease Epidemiology, London School of Hygiene and Tropical Medicine, London, WC1E 7HT, UK; 9 Department of Psychiatry, University of Oxford, Oxford, OX3 7JX, UK; 10 Institute for Human Development, Aga Khan University, Nairobi, 00100, Kenya; 11 Department of Paediatrics, University of Oxford, Oxford, OX3 9DU, UK

**Keywords:** Vitamin D deficiency, neurobehavioural outcomes, brain development, children, cognitive, motor, language, development

## Abstract

**Introduction:** Vitamin D plays an important role in brain development in experimental studies; however, the effect of vitamin D deficiency on child development remains inadequately characterized. We aimed to estimate the effects of vitamin D deficiency on neurobehavioural outcomes in children up to 18 years of age.

**Methods:** We searched PubMed, EMBASE, PsycINFO, Scopus, Cochrane Library, Web of Science and Open Grey for published studies up to 10th January 2020. We included all studies that assessed the effects of maternal or child vitamin D status or vitamin D supplementation on neurobehavioural outcomes in children. Study findings were synthesized qualitatively as the high level of heterogeneity in study populations and methodologies precluded a quantitative meta-analysis.

**Results:** Our search identified 5,633 studies, of which 31 studies with 31,375 participants from 18 countries were included in the systematic review. Of the studies identified, one was a randomised controlled trial (RCT) of vitamin D supplementation in children, while 30 were observational. The RCT (n=55) reported a beneficial effect of supplementation with lower doses compared to higher doses of vitamin D on motor development. Twelve mother-child studies (n=17,136) and five studies in children (n=1,091) reported an association between low maternal or child 25-hydroxyvitamin D levels and impaired neurobehavioural outcomes in children, while 15 mother-child studies (n=20,778) and eight studies in children (n=7,496) reported no association.

**Conclusions:** Although animal studies point to an effect of vitamin D deficiency on brain development, there are few studies on the effects of vitamin D deficiency on neurobehavioural outcomes in children and their findings are inconsistent. There is a need for well-conducted, adequately powered studies to further determine these effects in children.

**Registration:** PROSPERO ID CRD42018087619; registered on 15 February 2018.

## Introduction

Impaired neurobehavioural outcomes are common among children worldwide, especially in low- and middle-income countries (LMICs) ^1^. Approximately 81 million children below the age of five years have impaired cognitive and socioemotional development in LMICs globally and 44% of these children live in sub-Saharan Africa ^2^. Impaired child development is associated with poor educational achievement and subsequent poverty and poor health outcomes ^3^. Risk factors for impaired development include home environments lacking in stimulation, infections, malnutrition and micronutrient deficiencies ^3, 4^. Vitamin D deficiency and/or insufficiency is an important public health concern that affects approximately one billion people globally ^5, 6^. Evidence suggests that vitamin D deficiency is particularly widespread among children worldwide ^7– 9^.

**Figure 1.**
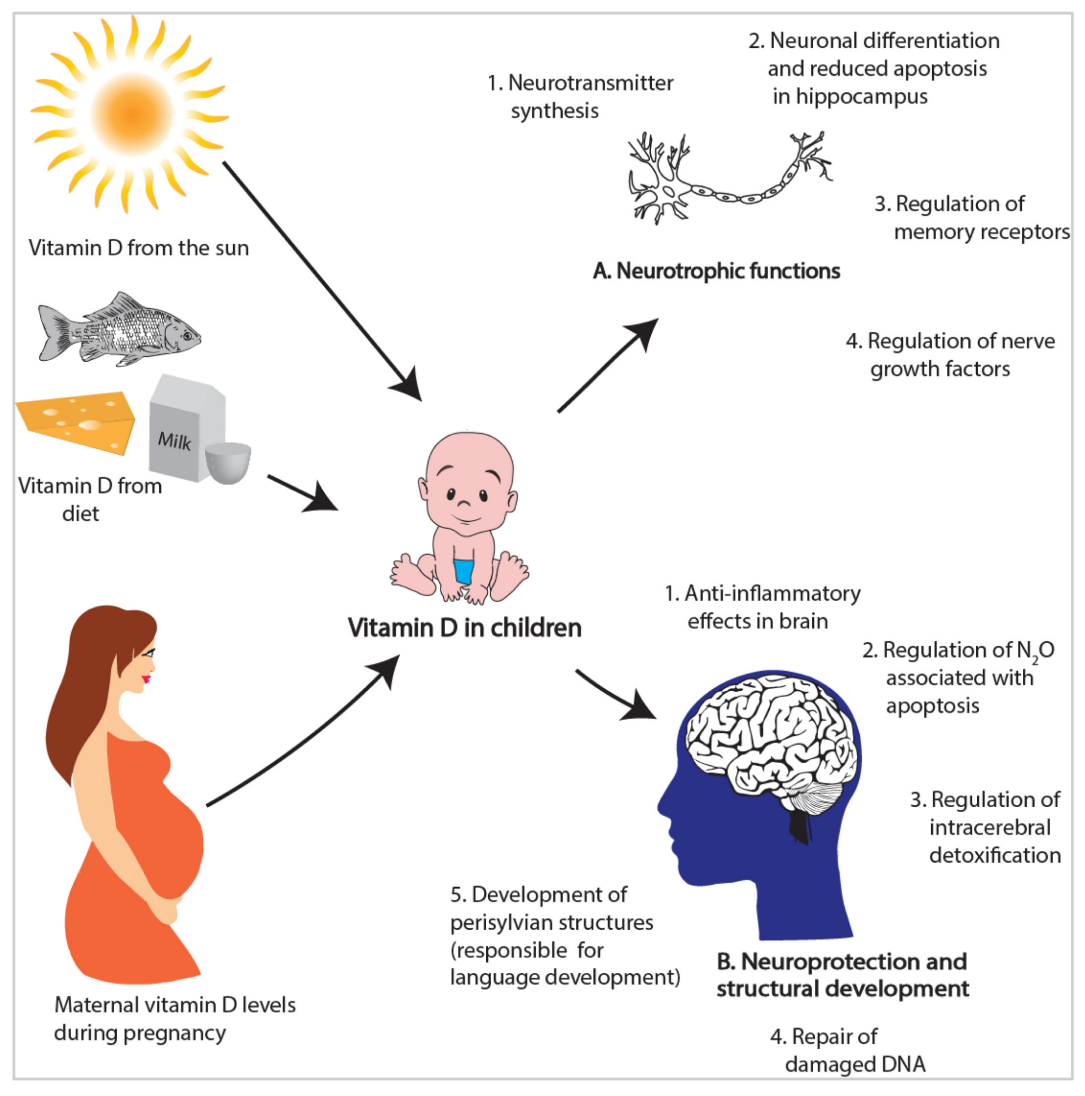
Factors influencing vitamin D status in children and potential roles of vitamin D in brain development ^11– 16, 20– 22^.

Vitamin D may be important for brain development, especially in the early years of life when the brain is developing rapidly and is sensitive to nutrient deficiencies ^10^. Animal and *in vitro* studies provide consistent evidence for an important role of vitamin D in brain development. Evidence includes indication of vitamin D signalling in the brain, including presence of vitamin D receptors (VDR), metabolites and enzymes responsible for vitamin D activation and inactivation ^11, 12^. Furthermore, animal studies show that vitamin D is important for neuronal differentiation and reducing apoptosis in the hippocampus, an area that is involved in language and memory ^13, 14^. Vitamin D is also important for neuroprotection and anti-inflammatory effects in the brain ^15, 16^ ( Figure 1). However, there are few epidemiological studies that have evaluated the effects of maternal or child vitamin D status on neurobehavioural outcomes.

In this systematic literature review, our objective was to estimate the effects of vitamin D deficiency and/or vitamin D supplementation on neurobehavioural outcomes in children. The outcomes of interest included cognitive and motor development, behaviour, intelligence, memory, school achievement and language. We further discuss the possible mechanisms by which vitamin D deficiency might influence child development from *in vitro* and animal studies.

## Methods

### Reporting guidelines

We used the Preferred Reporting Items for Systematic Review and Meta-analysis (PRISMA) guidelines ^17, 18^ and the Centre for Reviews and Dissemination (CRD) recommendations for undertaking reviews in healthcare ^19^. We registered the protocol on the PROSPERO database (registration number CRD42018087619).

### Search strategy and eligibility criteria

We searched PubMed, EMBASE, PsycINFO, Scopus, Cochrane Library and Web of Science for relevant studies published up to 10 ^th^ January 2020 without geographical or language limitations. We also searched the Open Grey database for unpublished studies. We further scanned reference lists of identified studies and previous systematic reviews for relevant studies. We applied a search strategy combining Medical Subject Heading terms for [vitamin D] AND [neurobehavioural outcomes] AND [children] and modified the search strategy as appropriate for each of the specific databases ( *Extended data*, file 1) ^18^.

We included studies if they (i) involved participants below 18 years of age or mother-child pairs; (ii) involved supplementation with vitamin D or measured 25(OH)D levels in children or in pregnant mothers; (iii) measured neurobehavioural outcomes in children including cognitive or motor development, intelligence quotient, attention, behaviour, school achievement or language. We excluded studies that were in older populations or only studied other outcomes apart from neurobehavioural outcomes. We also excluded reviews and case studies, commentaries, study protocols, reports and letters.

### Study selection, data extraction and quality appraisal

First, we screened titles and abstracts of all identified studies against the inclusion criteria. We then reviewed full texts for potentially relevant articles to determine eligibility for inclusion. We extracted data into an Excel spreadsheet with a list of variables determined *a priori* by the authors. Variables that were extracted for the review included the first author’s name and year of publication, study design, country, sample size, age at vitamin D and neurobehavioural assessment, neurobehavioural outcomes and tools used for evaluation, 25(OH)D cut-offs, and findings of the study. Study selection and data extraction was carried out by two reviewers independently (AMM and RMM) and then compared. Disagreements between reviewers were resolved through discussion. For this review, we defined severe vitamin D deficiency, vitamin D deficiency, insufficiency and sufficiency as 25(OH)D levels <25 nmol/L, 25–50 nmol/L, 50–75 nmol/L and >75 nmol/L respectively according to the Endocrine Society Practice Guidelines ^23^.

We used the Cochrane handbook criteria to assess the risk of bias for the randomized controlled trial (RCT) ^24^ and the Strengthening the Reporting of Observational Studies in Epidemiology (STROBE) checklist to assess for the quality of observational studies included in the review ^25^.

### Synthesis of included articles

The large degree of diversity in the study populations and methods necessitated qualitative synthesis for the studies. We grouped and discussed the findings by each neurobehavioural outcome examined and used tables to present a summary of the study characteristics and findings. We compared study findings based on when 25(OH)D levels were measured, assays used to measure 25(OH)D levels, definitions of vitamin D status and tools used to assess neurobehavioural outcomes. For consistency, we converted all units of 25(OH)D measurements into nmol/L.

## Results

### Study selection

**Figure 2.**
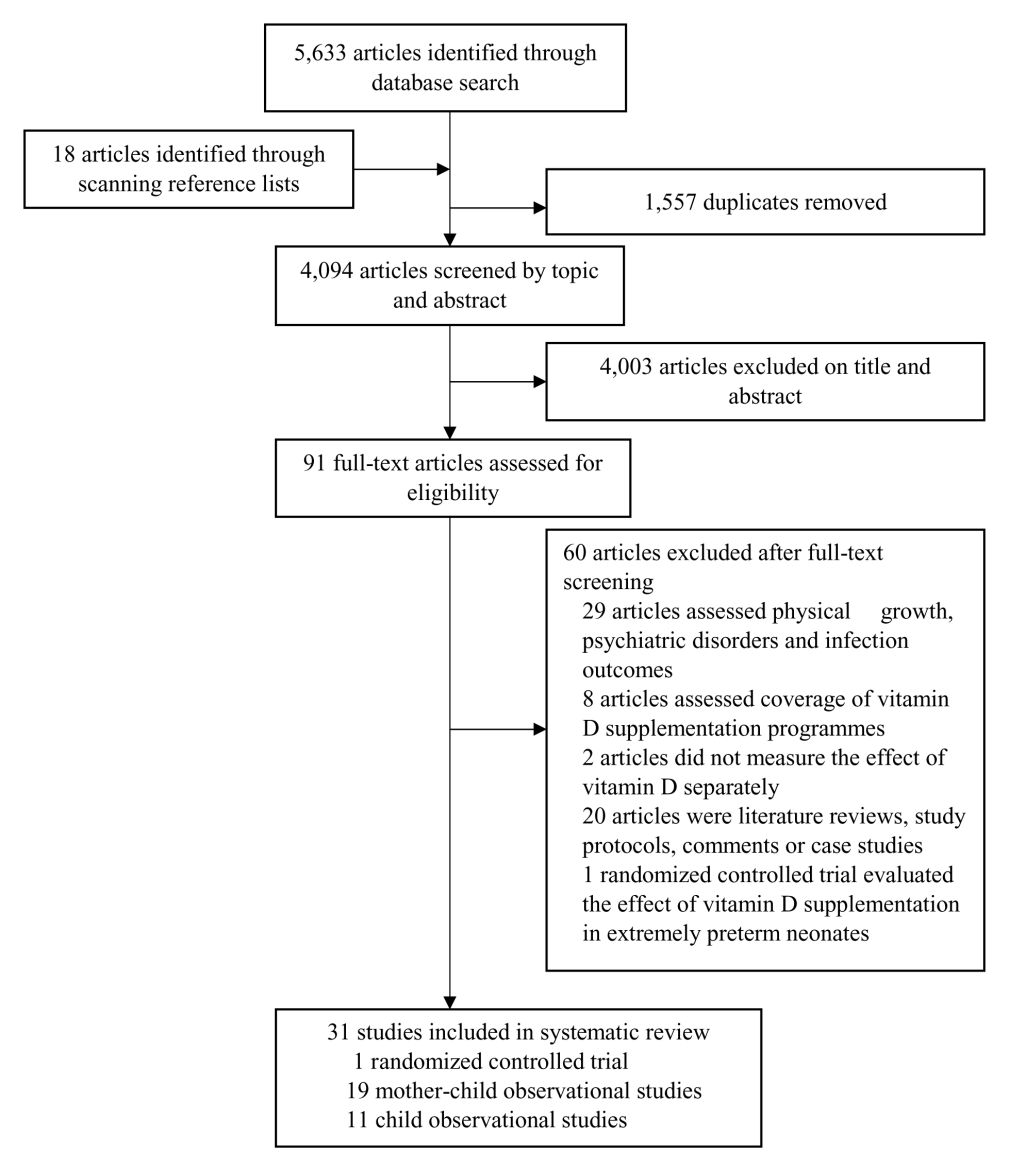
PRISMA flow chart showing the selection process for studies included in the review.

Our search identified 5,633 articles and a further 18 articles were identified from scanning through reference lists of relevant articles ( Figure 2). We removed 1,557 duplicate articles. After screening abstracts and titles, we excluded 4,003 articles that were irrelevant to our study and retained 91 papers for full review. We excluded 29 studies because the outcomes of interest were physical growth, psychiatric disorders or infections, eight because they assessed coverage of vitamin D supplementation programmes and two RCTs because the effect of vitamin D status on child development was not measured separately from the effects of other micronutrients. We excluded one RCT as it evaluated the effect of vitamin D supplementation on cognitive development in extremely preterm neonates ^26^. We excluded an additional 20 articles as they were reviews, case studies, commentaries or study protocols.

### Study characteristics and outcomes

**Figure 3.**
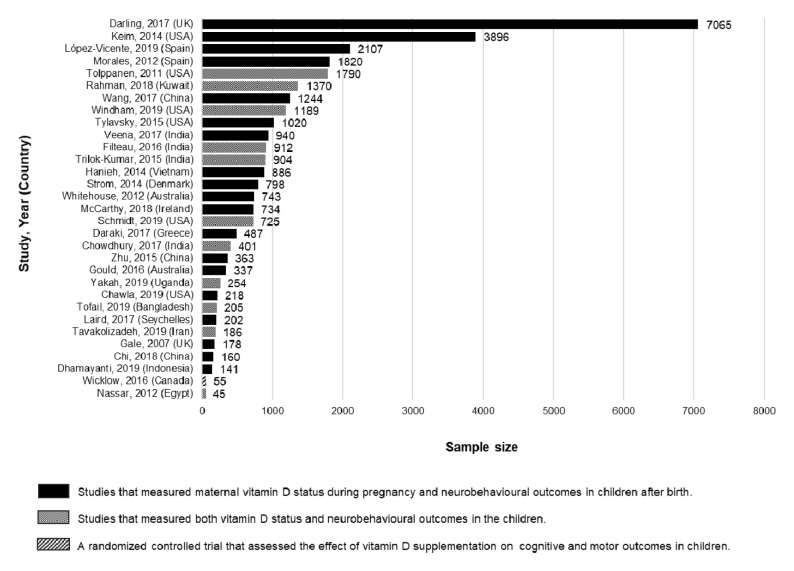
Sample sizes and types of studies included in the review.

We included one vitamin D supplementation trial and 30 observational studies published between 2007 and 2019 in this review. Of the 30 observational studies, 19 evaluated the effect of maternal vitamin D status during pregnancy and 11 the effect of a child’s vitamin D status on neurobehavioural outcomes in children. Of the 11 studies in children, three were cohort studies, two were case-control studies and six were cross-sectional studies; 19 studies were carried out in high-income countries and 13 in low and middle-income countries. The sample sizes varied across the studies, from 45 to 7065 children ( Figure 3). The studies evaluated a range of neurobehavioural outcomes using a variety of assessment tools. In 21 studies, neurobehavioural assessments were conducted by psychologists or trained research or clinical staff, seven studies relied on caregiver reports, while three studies did not provide information on assessors. The studies used varying definitions for vitamin D status and different assays to quantify 25(OH)D levels ( Table 1). All five studies that measured 25(OH)D levels in cord blood did not provide information on the sampling procedures. The characteristics and results of the included studies are shown in Table 1. The RCT showed a low risk of bias *(Extended data* file 2) ^18^, although the small sample size (n=55) is likely to have limited its power and external validity. All observational studies adjusted for multiple potential confounding factors except one study in which it was unclear whether they adjusted for any confounders ^27^. Limitations of the observational studies included a lack of description about loss to follow-up, and many did not report the reasons for non-participation and the number of participants missing data for each variable ( *Extended data*, file 3) ^18^.

**Table 1. T1:** Summary of studies assessing the effect of vitamin D status on child development: characteristics and findings.

Author, year (Country)	Participants	Age at vitamin D measurement (gestational or in child)	Age at neuro- assessment in child	Study design	Domain (tool)	Definition for vitamin D status	25(OH)D quantification method	Results
Vitamin D supplementation randomized controlled trials								
Wicklow, 2016 (Canada) ^45^	55 infants	3 and 6 months	6 months	RCT: 3 arms given 400 IU (n=19), 800 IU (n=18) or 1,200 IU (n=18) vitamin D3/day for 5.5 months	Motor (AIMS)	VDS: >75 nmol/L	LC-MS/MS	Infants given 400 IU of vitamin D had higher motor scores (percentile scores (SD)= 44.7 (27.7)) compared to those who received 800 IU and 1,200 IU (23.6 (13.9) and 25.6 (17.7) respectively, p <0.05).
Studies that measured maternal 25(OH)D levels during pregnancy and neurobehavioural outcomes in children after delivery								
López-Vicente, 2019 (Spain) ^46^	2107 mother-child pairs	Mean (SD) 13.3 (1 ^st^ to 2 ^nd^ trimester	4 time-points: 5,8,14 and18 years	Cohort	Social competence Total behavioural problems (CBCL, SDQ)	VDD: <50 nmol/L VDI: 50–74.9 nmol/L VDS: ≥75 nmol/L	HPLC	Maternal 25(OH)D levels >75 nmol/L associated with improved social competence scores compared to levels <50 nmol/L (β=2.14, 95% CI= 0.47, 3.82). No association with total behavioural problems.
Dhamayanti, 2019 (Indonesia) ^37^	141 mother- child pairs	11 to 14 weeks (1 ^st^ trimester)	3 time-points: 3, 6 and 12 months	Cohort	Motor Cognition (ASQ-3)	VDD: <25 nmol/L	ELFA	Maternal 25(OH)D levels <25 nmol/L associated with impaired motor development at 3 and 12 months. No association with cognition.
Chawla, 2019 (USA) ^47^	218 mother- child pairs	1 ^st^ and 2 ^nd^ trimester	12 to 24 months	Cohort	Behaviour (ITSEA questionnaire)	25(OH)D levels were divided into 4 quartiles (exact values unspecified)	EIA	Lower maternal quartiles of 25(OH)D associated with less favourable behavioural scores among white and Hispanic, but not black infants.
Chi, 2018 (China) ^31^	160 mother- child pairs	28 weeks (2 ^nd^ trimester)	6 months	Cohort	Mental Psychomotor (BSID-III)	VDD: <50 nmol/L VDS: >50 nmol/L	ECLIA	Maternal 25(OH)D <50 nmol/L associated with lower mental (OR=2.77, 95% CI: 1.44, 5.35, p=0.002) and psychomotor scores (OR=2.08, 95% CI: 1.07, 4.04, p=0.032)
McCarthy, 2018 (Ireland) ^38^	734 mother- child pairs	Two time- points: 15 weeks (2 ^nd^ trimester) and cord blood	5 years	Cohort	IQ Behaviour (KBIT-2, CBCL)	VDD: <30 nmol/L VDI: 30–50 nmol/L VDS: ≥ 50 nmol/L	LC-MS/MS	No association with IQ or behaviour.
Darling, 2017 (UK) ^32^	7065 mother-child pairs	Median 29·6 (12·7–33·3) (1 ^st^ to 3 ^rd^ trimester)	Motor, socio- development at 6 to 42 months Behaviour at 7 years IQ at 8 years Reading ability at 9 years	Cohort	Motor Socio-development and communication Behaviour IQ Reading ability (SDQ, WISC)	VDD: <50 nmol/L VDS: >50 nmol/L	HPLC	Maternal 25(OH)D <50 nmol/L associated with lower gross-motor (OR=1.20, 95% CI: 1.03, 1.40, p=0.02), fine-motor (OR=1.23, 95% CI: 1.05, 1.44, p=0.01) and social development (OR=1.20, 95% CI: 1.01,1.41, p=0.04). No association with IQ or reading ability.
Veena, 2017 (India) ^35^	940 mother- child pairs	28 to 32 (3 ^rd^ trimester)	9–14 years	Cohort	Cognitive Memory Language (KABC-II, WISC)	VDD: <50 nmol/L VDS: >50 nmol/L	RIA	No association with cognitive, memory or language development.
Wang, 2017 (China) ^33^	1244 mother-child pairs	Cord blood	2 years	Cohort	Motor Cognitive Behaviour (ASQ)	VDD: <50 nmol/L VDI: 50-74.9 nmol/L VDS: ≥75 nmol/L	LC-MS/MS	No association with motor, cognitive or behavioural development.
Laird, 2017 (Seychelles) ^39^	202 mother- child pairs	Maternal blood samples collected 1 day after delivery	5 years	Cohort	Cognitive Behaviour Language (FT, PLS, WSAT, CBCL, KBIT)	VDS: ≥50 nmol/L VDI: 30–49.9 nmol/L VDD: <30 nmol/L	LC-MS/MS	No association with cognitive, language or behavioural development.
Daraki, 2017 (Greece) ^40^	487 mother- child pairs	13±2.4 weeks (1 ^st^ trimester)	4 years	Cohort	Cognitive Motor Behaviour (MSCA, SDQ)	Tertiles: <38.4 nmol/l; 38.4–50.7 nmol/l; >50.7 nmol/l.	CLIA	Maternal 25(OH)D levels >50.7 nmol/l associated with higher behavioural scores compared to maternal 25(OH)D <38.4 nmol/l. No association with cognitive or motor development.
Gould, 2016 (Australia) ^41^	337 mother- child pairs	Cord blood	Cognitive, motor, language, behaviour and socio-emotional at 18 months Language at 4 years	Cohort	Cognitive Motor Behaviour Language (BSID-III, DAS II, CELF-P2)	VDD: <25 nmol/L VDI: 25–50 nmol/L VDS: >50 nmol/L	LC-MS/MS	Each 10 nmol/L increase in cord blood 25(OH)D was associated with an increase in mean language scores of 0.60 points at 18 months (95% CI: 0.04, 1.17, P = 0.04) and 0.68 points at 4 years (95% CI: 0.07, 1.29, P = 0.03). No association with cognition, motor, behaviour or socio-emotional development.
Zhu, 2015 (China) ^30^	363 mother- child pairs	Cord blood	16–18 months	Cohort	Mental Psychomotor (BSID-CR)	Quintiles: 5.56-20.8, 20.9- 30.9, 31.0-39.8, 39.9-51.0, 51.3-111 nmol/L	RIA	Cord blood 25(OH)D levels of 5.56 to 20.8 nmol/L associated with a reduction in mental and psychomotor scores (-7.60 ,95% CI: -12.4, -2.82; P=0.002 and -8.04, 95% CI: 212.9, 23.11; P=0.001 points respectively) compared to the reference category (25(OH)D levels 39.9-51.0 nmol/L). Levels of 51.3 to 111 nmol/L associated with a reduction in psychomotor scores -12.3 (95% CI: 217.9, 26.67; P<0.001) points) compared to the reference category.
Tylavsky, 2015 (USA) ^34^	1020 mother-child pairs	2 ^nd^ trimester	2 years	Cohort	Cognitive Language (BSID III)	Categories; <49.9, 50.00- 74.9, ≥75 nmol/L	EIA	Each 25 nmol/L increase in maternal 25(OH)D was associated with an increase in scaled receptive language scores of 0.24 points (P = 0.017). No association with scaled cognitive or expressive language scores.
Hanieh, 2014 (Vietnam) ^36^	886 mother- child pairs	32 weeks (3 ^rd^ trimester)	6 months	Cohort	Cognitive Motor Behaviour Language (BSID III)	VDD: <37.5nmol/L VDI: ≥37.5-75 nmol/L VDS: ≥75 nmol/L	LC-MS/MS	Maternal 25(OH)D < 37.5 nmol/L associated with a reduction in language scores (mean difference: 23.48; 95% CI: 25.52,1.44). No association with cognitive, motor, or behaviour development.
Keim, 2014 (USA) ^28^	3896 mother-child pairs	Two time- points: ≤26 weeks (2 ^nd^ trimester) and Cord blood	Cognitive and motor at 8 months IQ and behaviour at 4 and 7 years School achievement at 7 years	Cohort	IQ Motor Behaviour School achievemen (BSID, SBIS, WISC, WRAT)	Categories: <25, 25–50, 50–75, ≥75 nmol/L	LC-MS/MS	Each 5 nmol/L increase in both maternal and cord blood 25(OH)D associated with a slight increase in IQ scores of 0.10 points (95% CI: 0.00, 0.19) at 7 years of age. No association with motor, school achievement or behaviour development.
Strøm, 2014 (Denmark) ^48^	798 mother- child pairs	30 weeks (2 ^nd^ trimester)	15–16 years	Cohort	School achievement (Exam scores)	VDD: <50 nmol/L VDS: ≥50–75 nmol/L Optimal levels: ≥75–125 nmol/L	LC-MS/MS	No association between maternal 25(OH)D and school achievement.
Morales, 2012 (Spain) ^29^	1820 mother-child pairs	11.6% assessed in 1 ^st^ trimester, 88% in 2 ^nd^ trimester and <1% in 3 ^rd^ trimester	11–23 months	Cohort	Mental Psychomotor (BSID)	VDD: <50 nmol/L VDS: >75 nmol/L	HPLC	Each 25 nmol/L increase in maternal 25(OH)D associated with 0.79 (95% CI: 0.14 to 1.45) and 0.88 (95% CI: 0.22 to 1.54) points increase in mental and psychomotor scores, respectively.
Whitehouse, 2012 (Australia) ^49^	743 mother- child pairs	18 weeks (2 ^nd^ trimester)	2 years	Cohort	Language Behaviour (CBLC, PPVT-R)	VDD: <46 nmol/L	EIA	Maternal 25(OH)D levels <46 nmol/L were associated with impaired language development compared to levels >72 nmol/L (OR=1.97; 95% CI 1.00 to 3.92; P < 0.05). No association with behavioural development.
Gale, 2007 (UK) ^42^	178 mother- child pairs	28–42 weeks (3 ^rd^ trimester)	9 years	Cohort	Cognitive (WASI)	VDS: >50 nmol/L VDI: 27.5–50 nmol/L VDD: <27.5 nmol/L	RIA	No association with cognitive development.
Studies that measured 25(OH)D levels and neurobehavioural outcomes in children								
Tofail, 2019 (Bangladesh) ^50^	205 children	7 months	6–8 months	Cross-sectional	Cognitive Motor Language Behaviour (BSID III, parental reports)	VDS≥50 nmol/L VDD<50 nmol/L	ELISA	Children with VDD had lower behavioural scores (activity: β ±SE 1.64±0.78; 95% CI 0.10, 3.18; p=0.037) and soothability (2.02±0.70; 0.64, 3.41; p=0.004) compared to the VDS group. No association with cognitive, motor and language development.
Yakah, 2019 (Uganda) ^51^	254 children (3 groups: perinatally HIV-infected (PHIV), exposed- uninfected (HEU), or unexposed- uninfected (HUU))	6–10 years	3 time-points: at birth, 6 and 12 months	Cohort	Behaviour BASC-3	VDD: <50 nmol/L VDI: 50-62 nmol/L VDS: >62 nmol/L	HPLC-MS/MS	VDD associated with poor behavioural development in perinatally HIV exposed but uninfected and HIV unexposed uninfected (HUU).
Windham, 2019 (USA) ^52^	1189 children (753 cases with developmental disability and 436 controls from the general population)	New-born stored blood spots	4.5– 9 years	Case-control	IQ (Intellectual disability)	VDS: ≥ 75nmol/L VDI: 50–74nmol/L VDD: <50 nmol/L	LC-MS/MS	No association with IQ.
Schmidt, 2019 (USA) ^53^	725 children (491 cases with developmental disability and 234 controls from the general population)	New-born stored blood spots.	24–60 months	Case-control	Cognitive (MSEL, VABS)	VDS: ≥75 nmol/L VDI: 50–75 nmol/L VDD: <50 nmol/L	LC-MS/MS	No association with cognitive development.
Tavakolizadeh, 2019 (Iran) ^27^	186 children	12 months	12 months	Cross-sectional	Gross motor (Assessed by paediatrician based on achievement of pre-selected milestones)	VDS: >75 nmol/L VDI: 50– 74 nmol/L VDD: < 50 nmol/L	ELISA	VDD associated with impaired gross motor ability compared to VDS (OR=3.9, 95% CI=1.9-8.4, P<0.001).
Rahman, 2018 (Kuwait) ^54^	1,370 adolescents	11–16 years	11–16 years	Cross-sectional	Cognitive (RSPM), School achievement (Exam scores)	VDS: ≥75 nmol/L VDI: 50–75 nmol/L VDD: <50 nmol/L Severe VDD: <25 nmol/L	LC-MS/MS	No association with cognitive development or school achievement.
Chowdhury, 2017 (India) ^44^	401 pre- school children	6–30 months	12–36 months	Cohort	Cognitive Motor Personal- social (ASQ-3)	VDD: <25 nmol/L	ECLIA	No association with cognitive, motor or personal social development.
Filteau, 2016 (India) ^55^	912 school children	5 years	5 years	Cross-sectional	Gross motor (ASQ-3)	VDD: <25 nmol/L Borderline: 25–50 nmol/L VDS: >50 nmol/L	RIA	No association with gross motor development.
Trilok-Kumar, 2015 (India) ^56^	904 children	2 time-points: 6 months, 3–6 years	3–6 years	Cohort (follow up of a vitamin D supplementation trial in infancy)	Gross motor (ASQ-2)	VDD: <50 nmol/L	RIA	No association with gross motor development.
Nassar, 2012 (Egypt) ^43^	45 school children	School age	School age	Cross-sectional	Cognitive (WISC, BVRT)	VDD: <27.5 nmol/L VDI: 27.5–37.5 nmol/L VDS: 37.5–77.5 nmol/L	HPLC	VDS associated with improved cognitive and school achievement compared to VDD.
Tolppanen, 2011 (USA) ^57^	1790 adolescents	12–16.9 years	12–16.9 years	Cross-sectional	Cognitive (WISC-R, WRAT)	Quartiles: 8.7–49.2, 49.4–65.6, 65.9–82.1, 82.4–205.2 nmol/L	RIA	No association with cognitive development.

AIMS, Alberta Infant Motor Scale; ASQ-3, Ages and Stages Questionnaire-3; BASC-3, Behavioural Assessment System for Children; BSID, Bailey scales of infant development; BSID-CR, Bayley Scales of Infant Development-China Revision; BSID-III, Bayley Scales of Infant Development-version 3; BVRT, Benton's Visual Retention Test; CBLC, Child Behaviour checklist; CELF-P2, Clinical Evaluation of Language Fundamentals Preschool; Second Edition; DAS-II, Differential Ability Scales Second Edition; FT, Finger tapping; LC-MS/MS, liquid chromatography-tandem mass spectrometry; RIA, radioimmunoassay; HPLC, high performance liquid chromatography; HPLC-MS/MS, high performance liquid chromatography tandem mass spectrometry; ECLIA, electro-chemiluminesce inmmunoassay; ELISA, enzyme-linked immunosorbent assay; EIA, enzyme immunoassay; CLIA, chemiluminescence immunoassay; PLS, Preschool Language Scale-Revised Edition; MSCA, McCarthy Scales of Children Abilities; WSAT, Woodcock-Johnson Scholastic Achievement Test; KBIT, Kaufman Brief Intelligence Test; RSPM, Raven’s Standard Progressive Matrices; PPVT-R, Peabody Picture Vocabulary Test-Revised; SBIS, Stanford-Binet Intelligence Scale; WASI, Wechsler Abbreviated Scale of Intelligence; WISC, Wechsler Intelligence Scale for Children; WISC-R, Wechsler Intelligence Scale for Children-revised; WRAT, Wide Range Achievement Test; SDQ, Strengths and Difficulties Questionnaire; KABC-II, Kaufman Assessment Battery for Children-2nd edition; MSEL, Mullen Scales of Early Learning; VABS, Vineland Adaptive Behaviour Scales. IU, international units; VDD, vitamin D deficiency; VDI, vitamin D insufficiency; VDS, vitamin D sufficiency; β, Beta coefficient.

### Cognitive development

A total of 15 studies, including 19,810 mother-child pairs evaluated the effect of vitamin D status during pregnancy on cognitive development in children. Of these, four mother-child studies (n=6,239) found a beneficial effect of higher maternal 25(OH)D levels on cognitive development and 11 studies (n=13,234) reported no association. In a large study of 3,896 American mother-child pairs, higher maternal 25(OH)D levels were associated with higher intelligence quotient (IQ) scores in children at 7 years of age ^28^. Likewise, in a study of 1,820 Spanish mother-child pairs, maternal 25(OH)D levels >75 nmol/L were associated with higher cognitive scores in children aged 11–23 months compared to maternal 25(OH)D levels <50 nmol/L ^29^. Another study of 363 Chinese mother-child pairs reported that cord blood 25(OH)D levels between 5.56 and 20.8 nmol/L were associated with lower cognitive scores in 16–18 month-old children than levels between 39.9 and 51 nmol/L ^30^. Similarly, a study of 160 Chinese mother-child pairs found that maternal 25(OH)D levels <50 nmol/L were associated with low cognitive scores in six-month-old children ^31^.

However, a large study of 7,065 mother-child pairs in the UK reported no association between maternal 25(OH)D levels and cognition in eight-year-old children ^32^. Other studies in 1,020 American and 1,244 Chinese mother-child pairs also found no association between maternal 25(OH)D levels during pregnancy and cognition in children aged two years or below ^33, 34^. Two mother-child studies in India (n=940) and Vietnam (n=886) also found no association between maternal 25(OH)D levels during pregnancy and cognition in children aged six months to 14 years ^35, 36^. Similarly, in six other studies of 2,079 mother-child pairs, maternal 25(OH)D levels were not associated with cognitive development after birth ^37– 42^.

Seven observational studies measured both 25(OH)D levels and cognitive development in children. One small cross-sectional study (n=45) in Egyptian school children reported that those with 25(OH)D levels of 37.5–77.5 nmol/L had improved cognitive function compared to those with 25(OH)D levels <27.5 nmol/L ^43^. However, a cohort study in 401 Indian children found no association between vitamin D status at six to 30 months of age with cognitive development at 12 to 36 months of age ^44^. Additionally, two case-control studies of 1,244 children with neurodevelopmental disabilities and 670 controls with normal development in the USA reported no association between vitamin D status and cognitive development at two to nine years of age ^52, 53^. Likewise, a cross-sectional study of 1,790 American adolescents aged 12 to 16.9 years did not find an association with cognitive development ^57^. Similarly, two cross-sectional studies, one of 1,370 Kuwaiti adolescents aged 11 to 16 years old and another of 205 Bangladeshi children aged six to eight months reported no association between vitamin D status and cognitive development ^50, 54^.

### Motor development

One small vitamin D supplementation trial in 55 Canadian children aged three to six months evaluated the effect of vitamin D in doses of 400 international units (IU), 800 IU and 1200 IU on gross motor skills. The study did not include a placebo arm. They reported that supplementation with 400 IU vitamin D was beneficial for gross motor development in infants compared to supplementation with 800 or 1200 IU of vitamin D ^45^.

A total of 10 studies including 11,503 mother-child pairs examined the association between motor development and vitamin D status. In five mother-child studies (n=9,549), high maternal 25(OH)D levels were associated with improved motor development; however, five studies (n=6,850) found no association. A large study of 7,065 mother-child pairs in the UK reported a trend of improved gross and fine motor scores in 18–30 month-old children with increasing maternal 25(OH)D levels ^32^. Another study of 1,820 Spanish mother-child pairs, similarly showed that maternal 25(OH)D levels >75 nmol/L were associated with higher motor scores in infancy compared to levels <50 nmol/L ^29^. A study of 160 Chinese mother-child pairs showed that vitamin D deficient (25(OH)D levels <50 nmol/L) mothers were more likely to have children with lower psychomotor development scores at six months of age compared to mothers without deficiency ^31^. Another study of 141 Indonesian mother-child pairs reported that maternal 25(OH)D levels <25 nmol/L were associated with impaired motor development in children at three and 12 months of age ^37^. Interestingly, a study of 363 Chinese mother-child pairs reported an inverted u-shaped trend of association with lower motor scores in toddlers that had cord blood 25(OH)D levels between 5.56 and 20.8 nmol/L and between 51.3 and 111 nmol/L compared to the reference category (levels 31.0 to 39.8 nmol/L) ^30^. However, two large mother-child studies in the USA (n=3,896) and China (n=1,244) and smaller studies in Vietnam (n=886), Greece (n=487) and Australia (n=337) reported no association between 25(OH)D levels during pregnancy and motor development in children aged between six months to four years ^28, 33, 36, 40, 41^.

Five studies assessed both 25(OH)D levels and motor development in children. A cross-sectional study among 186 Iranian children reported that 25(OH)D levels >75 nmol/L were associated with improved gross motor ability compared to 25(OH)D levels <50 nmol/L at one year of age ^27^. However, a cohort study in 401 Indian children reported no association between vitamin D status measured at six to 30 months and motor development at 12 to 36 months ^44^. Another cohort study in 904 Indian children found that vitamin D status measured at six months and at three to six years was not associated with gross motor development at three to six years of age ^56^. Likewise, two cross-sectional studies, one in 912 five-year-old Indian children and another in 205 Bangladeshi infants aged six to eight months reported no association with motor development ^50, 55^.

### Language development

The association between maternal 25(OH)D levels and language development was evaluated in six studies including a total of 4,128 mother-child pairs. Four studies, including a total of 2,986 mother-child pairs, reported a positive association between high maternal 25(OH)D levels and improved language development, while two other studies including 1,142 mother-child pairs reported no association. A study of 1,020 American mother-child pairs reported that maternal 25(OH)D levels ≥75 nmol/L were positively associated with receptive language but not expressive language in two-year-old children ^34^. Likewise, a study of 886 Vietnamese mother-child pairs reported that six-month-old infants born to women with 25(OH)D levels <37.5 nmol/L had lower language scores compared to those with 25(OH)D levels ≥75 nmol/L ^36^. Similarly, in a study involving 743 mother-child pairs, children of mothers with 25(OH)D levels <46 nmol/L were almost two times more likely to have language impairment compared to those of women with 25(OH)D levels >72 nmol/L at two years of age ^49^. In another study of 337 Australian mother-child pairs, higher cord blood 25(OH)D levels were associated with an increase in mean language scores at 18 months and four years ^41^. However, two studies of 1,142 mother-child pairs reported no association between vitamin D status and language development in children aged five to 14 years ^35, 39^. A single cross-sectional study in 205 Bangladeshi infants aged six to eight months reported a positive association between 25(OH)D levels >50 nmol/L in children and language development compared to levels <50 nmol/L ^50^.

### Behavioural development

The association between maternal 25(OH)D levels and behavioural development was evaluated in 11 mother-child studies. Four mother-child studies (n=7,770) reported an association between high 25(OH)D levels during pregnancy and improved behavioural development in children; however, eight studies (n=10,149) found no association. A large study of 7,065 mother-child pairs in the UK reported an increasing trend of social development scores with increasing maternal 25(OH)D levels in children at 42 months of age ^32^. Another study of 487 mother-child pairs in Greece reported that maternal 25(OH)D levels >50.7 nmol/l were associated with better behavioural scores compared to 25(OH)D levels <38.4 nmol/l ^40^. Likewise, a study of 218 mother-child pairs in the USA reported that low maternal 25(OH)D levels (unspecified values) were associated with impaired behavioural development among white and Hispanic, but not black, infants at one to two years of age ^47^. A study of 2,107 mother-child pairs reported a positive association between maternal 25(OH)D levels >75 nmol/L and improved social competence scores compared to levels <50 nmol/L; however, they found little association between maternal 25(OH)D levels and total behavioural problems ^46^. Two large mother-child studies in the USA (n=3,896) and China (n=1,244) found no association between maternal 25(OH)D levels and behaviour in children aged two to 18 years ^28, 33^. Two other studies of 1,080 mother-child pairs in Australia reported no association between maternal 25(OH)D levels and behaviour in 18–24 month-old children ^41, 49^. Likewise, three mother-child studies in Vietnam (n=886), Ireland (n=734) and Seychelles (n=202) reported no association between maternal 25(OH)D levels and behaviour in children aged six months to five years ^36, 38, 39^.

Three studies measured both 25(OH)D levels and behavioural development in children. A cohort study of 254 Ugandan children found an association between 25(OH)D levels <50 nmol/L at enrollment, at six to 10 years of age, and poor behavioural outcomes measured at enrollment, and at six and 12 months after enrollment, among children who were perinatally exposed to HIV but were uninfected and unexposed HIV negative children ^51^. A cross-sectional study of 401 Indian children found that children with 25(OH)D levels <25 nmol/L had lower scores on the personal-social sub-scale at six to 36 months ^44^, and a cross-sectional study in 205 Bangladeshi children aged six to eight months reported an association between 25(OH)D levels <50 nmol/L and impaired parent-reported behaviour ^50^.

### School achievement

Two mother-child studies and two cross-sectional studies evaluated the effect of vitamin D status on school achievement. The two mother-child studies, including a total of 4,694 mother-child pairs, observed no association between maternal vitamin D status and children’s educational achievement ^28, 48^. The two cross-sectional studies reported conflicting findings. A study of 45 Egyptian children reported that 25(OH)D levels between 37.5 and 77.5 nmol/L were associated with improved school achievement compared to 25(OH)D levels <27.5 nmol/L ^43^, while a study of 1,370 Kuwaiti adolescents reported no association between vitamin D status and school achievement ^54^.

### Studies that assessed neurobehavioural outcomes during infancy

Six observational studies assessed neurobehavioural outcomes during infancy and of these studies, five reported associations between maternal or child 25(OH)D levels and neurobehavioural outcomes, while one study reported no associations. In contrast, 24 studies assessed neurobehavioural outcomes in children above one year of age and of these, 11 studies reported associations between maternal or child 25(OH)D levels and neurobehavioural outcomes, while 13 studies reported no associations.

### Comparing findings based on the timing, assays and cut-offs for 25(OH)D levels and neuroassessment tools

Six out of eight studies that measured maternal 25(OH)D levels in the 1 ^st^ or 2 ^nd^ trimester reported associations with neurobehavioural outcomes in contrast to three out of eight studies that measured 25(OH)D levels in the 3 ^rd^ trimester or in cord blood. Three out of seven studies that measured 25(OH)D levels in infancy and two out five studies that measured levels in children above one year reported associations with neurobehavioural outcomes ( Table 1). The most commonly used assay for measuring 25(OH)D levels was liquid chromatography-tandem mass spectroscopy (LC-MS/MS) ( Table 1) with four out of 11 studies that used LC-MS/MS reporting associations between maternal or child 25(OH)D levels and neurobehavioral outcomes, while 13 out of 20 studies that used other assays reported associations. The majority of studies (n=15) defined vitamin D deficiency as 25(OH)D levels <50 nmol/L and six of these studies reported associations with neurobehavioural outcomes. Six studies defined vitamin D deficiency as 25(OH)D levels <30 nmol/L or <25 nmol/L and of these two reported associations with neurobehavioural outcomes ( [Table T1]). The most common tool used to assess neurobehavioural outcomes was the Bayley Scales of Infant Development (BSID) and six out of eight studies that used the BSID reported associations between 25(OH)D levels and neurobehavioural outcomes ( Table 1).

## Discussion

In this systematic review, we found inconclusive evidence for the effects of maternal or child vitamin D status on neurobehavioural outcomes in children below 18 years of age. A small RCT (n=55) reported that infants given lower vitamin D doses had improved motor development compared to those given higher vitamin D doses suggesting that higher doses of vitamin D might impair motor development. Similarly, evidence from observational studies was conflicting. Twelve studies of 17,136 mother-child pairs and five studies in of 1,091 children reported an association between low maternal or child 25(OH)D and impaired neurobehavioural outcomes in children. However, 15 studies of 20,778 mother-child pairs and eight studies of 7,496 children found no association between vitamin D status and neurobehavioural outcomes.

We found conflicting evidence for the effects of vitamin D status on cognitive development. Overall, we found little evidence for a detrimental effect of low maternal or child 25(OH)D levels on cognitive development. Lack of association might be explained by the adequate baseline levels of 25(OH)D observed in some of the study populations. Of the 17 observational studies that reported no association, 11 observed maternal or child mean or median (25(OH)D levels >50 nmol/L), while four of the five studies that reported an association observed sub-optimal 25(OH)D levels (mean or median levels <50 nmol/L). Also, it is possible that some of the neurodevelopmental assessment tools were not sensitive enough to detect subtle effects of vitamin D on cognition. For example, Tylavsky *et al.* suggest that the Bailey Scales of Infant Development used in their study could have failed to detect small changes in recognition memory, symbolic play, acuity, or information processing speed ^34^. Although there is little evidence in humans, animal and *in vitro* studies suggest that vitamin D influences cognitive development ^58– 60^. *In vitro* studies show that vitamin D receptors and cytochrome enzymes responsible for vitamin D metabolism are present in the cortex and other parts of the brain involved in cognition ^11^. Additionally, vitamin D in its active form (1,25(OH) _2_D) influences cytokine production which may affect neurotransmission and synaptic plasticity, in turn altering learning processes and cognition as observed in rats ^61^.

Similarly, findings for an effect of vitamin D status on motor development were mixed. More mother-child studies indicated adverse effects for lower maternal 25(OH)D levels on motor development compared to cross-sectional studies in children. Only one cross-sectional study reported an association between lower 25(OH)D levels in children and motor development but these findings were unadjusted for potential confounders ^27^. Three of the four studies that reported no association between child vitamin D status and motor function used the Ages and Stages Questionnaire-3 to assess gross motor development ^44, 55, 56^. This tool was designed to diagnose children with developmental abnormalities and may not be sensitive enough to detect small changes in motor function ^62^. How might maternal vitamin D status affect motor development? Maternal vitamin D deficiency has been associated with congenital rickets with apparent foetal bone disease and impaired bone quality postnatally ^63^. One study reported an inverted u-shaped association so that both lower (5.56 to 20.8 nmol/L) and higher (51.3 to 111 nmol/L) maternal 25(OH)D levels were associated with impaired motor development in children ^30^. This finding is supported by a small vitamin D supplementation trial in 55 infants, which found that a lower dose of vitamin D supplementation might be more beneficial for gross motor function compared to higher doses of vitamin D ^45^. However, the findings of this trial may be limited by the lack of a control arm that did not receive vitamin D supplementation. It is possible that excess levels of vitamin D may result in toxic and pro-inflammatory effects which could lead to impaired motor development ^64, 65^ and that an optimal 25(OH)D level may be required. Vitamin D status might also influence motor development through its role in neurotransmission through the dopaminergic system. Data from animal studies show that vitamin D deficiency is associated with alterations in the synthesis and turnover of dopamine, norepinephrine and dihydroxyphenylacetic acid in rats ^20, 66, 67^ and ventral midbrain dopaminergic neurons have been shown to play a crucial role in the regulation of motor processes ^66^.

We found evidence for associations between low vitamin D status and impaired language development in four out of six mother-child studies. In one study, impairment of language development was only observed when 25(OH)D levels fell below 37.5 nmol/L in late pregnancy, suggesting that language impairment is a feature of advanced vitamin D deficiency rather than insufficiency ^36^. However, two mother-child studies (n=1,142) reported no association between maternal vitamin D status and language scores in adolescence, but these studies did not account for the vitamin D status of the adolescents, and it is possible that detrimental effects may have been evident earlier in life, but not in adolescence ^35, 39^. A possible mechanism for the observed association could be that *in utero* exposure to low vitamin D concentrations during the second and third trimesters may affect the development of the perisylvian structures, which are responsible for language development in children as reported in structural neuroimaging studies ^21, 68^. Additionally, maternal vitamin D deficiency has been shown to alter the brain structure and genes that are involved in neuronal survival and speech and language development (brain-derived neurotrophic factor ( *Bdnf*) and forkhead box protein P2 ( *Foxp2*) genes, respectively) in mice ^20^.

We found mixed evidence for the effect of vitamin D deficiency on behaviour in children. Eight of 11 mother-child studies reported no association between maternal vitamin D status and behaviour. Of these studies, four relied on parent-reported behaviours only, which might be prone to bias, and only three relied on psychologist assessments or well-validated tools in evaluating behaviour. Additionally, it might be difficult to estimate the effect of maternal vitamin D status on behavioural outcomes in childhood since a wide variety of maternal, environmental and genetic risk factors may cause confounding. Animal and *in vitro* studies suggest that vitamin D status might influence behaviour possibly via the anti-inflammatory effects of vitamin D, which protect against maternal immune activation, which may affect foetal brain development and behavioural manifestations ^69^. Additionally, vitamin D deficiency is associated with alterations in the synthesis of serotonin, a neurotransmitter that is important for behavioural outcomes ^70^.

There may be many explanations for our findings of inconsistent evidence for the effects of hypovitaminosis D on child development. There were substantial differences in study design between studies with some assessing maternal and some children’s vitamin D status in relation to child development. Studies also had different inclusion criteria and definitions for vitamin D status and were also adjusted for different confounding variables. Serum or plasma 25(OH)D levels were quantified using a variety of routinely available assays with most studies using the gold standard of liquid chromatography–tandem mass spectrometry(LC-MS/MS). There was little difference in neurobehavioral outcomes in studies that used LC-MS/MS and studies that used other assays to measure 25(OH)D levels. The lack of standardized assays may result in large inter-method variability and errors limiting comparability between studies ^71^. Secondly, vitamin D status was measured at varying gestational ages in mothers and different ages in the children. Several studies that measured maternal 25(OH)D in the 1 ^st^ or 2 ^nd^ trimester, rather than the 3 ^rd^ trimester or in cord blood, reported associations between low maternal 25(OH)D and low developmental scores in the children ^28, 29, 31, 34, 49^, which might suggest a critical window for the impact of low 25(OH)D levels earlier in pregnancy when there is rapid brain development ^72^. There was little difference when comparing findings between studies that measured 25(OH)D levels during or after infancy. In addition, studies suggest that there might be optimal levels of vitamin D required for development with higher levels also being associated with impaired development ^30, 45^. Thirdly, the timing and tools used to assess neurobehavioural outcomes varied markedly between studies. The majority of studies that assessed neurobehavioural outcomes during infancy reported associations between maternal or child 25(OH)D levels and neurobehavioral outcomes compared to fewer than half of studies that assessed neurobehavioural outcomes after infancy. Putative effects might be more likely to be detected in earlier years of life and may also be influenced by 25(OH)D levels throughout childhood. Most studies used the Bayley Scales of Infant Development to assess neurobehavioral outcomes in children, with more than half of these studies reporting associations between 25(OH)D levels and neurobehavioral outcomes. However, comparisons based on the neuroassessment tools were limited as most studies used several tools to assess different neurobehavioural outcomes. Studies that measured 25(OH)D levels during pregnancy and child development did not account for the vitamin D status of children at the time of neuroassessment, which may also have influenced neurobehavioural outcomes. The application of findings from experimental studies to human beings may also be limited. For instance, animal experiments can create conditions of extremely low 25(OH)D levels which may not normally be found in humans.

We identified two previous systematic reviews and one meta-analysis of the effects of maternal vitamin D status on neurobehavioural outcomes in children. Consistent with our review, a systematic review of maternal micronutrient status (vitamin D, B12, iron and folate) and carbohydrate, protein and fat intake during pregnancy and cognitive development in childhood found limited and conflicting evidence for the effect of vitamin D status on cognitive development ^73^. However, of 38 eligible studies, this review included only three mother-child studies on vitamin D status and cognitive development, also included in our review ^29, 42, 49^. Another systematic review also found contradictory evidence for the effect of maternal vitamin D status during pregnancy on neurobehavioural outcomes and neuropsychological disorders in children ^74^. This review included only 10 human studies, four of which evaluated neuropsychological disorders and six neurobehavioural outcomes, but cognition, motor, language and behavioural outcomes were not evaluated ^74^. Another systematic review and meta-analysis of the neurodevelopmental effects of prenatal vitamin D reported an association between increased maternal 25(OH)D levels and improved cognition and reduced risk of autism and attention deficit and hyperactivity disorder (ADHD) ^75^. Unlike our review, they did not include studies that measured vitamin D status of children. Additionally, the findings of the meta-analysis may be limited by the different definitions for vitamin D status used across the included studies ^75^.

### Strengths and limitations

A key strength of our review is that it was very comprehensive, since we searched seven databases without geographic location, date or language limitations. We included one randomized controlled trial and observational studies assessing the effects of maternal and child vitamin D status on neurobehavioural outcomes. Also, we included children up to the age of 18 years, hence capturing child development through to adulthood. There were several limitations in this review. We found substantial heterogeneity in study populations and study methods including timing of vitamin D measurements, definitions for vitamin D status and tools used to measure neurobehavioural outcomes which made comparison of the studies difficult and precluded a quantitative meta-analysis of the studies. However, we did not observe marked differences when comparing findings in studies that defined vitamin D deficiency as 25(OH)D levels <50 nmol/L with those that defined vitamin D deficiency as 25(OH)D levels <30 nmol/L or <25 nmol/L. There was only one small vitamin D supplementation trial and few studies measured 25(OH)D levels in children. We identified another small RCT (n=70) in the USA which reported no beneficial effect of vitamin D supplementation on cognition at two years of age ^26^, but we excluded this study as it included extremely preterm neonates, who are at a higher risk of neurodevelopmental delays compared to children born at full term ^76^. We also found a relatively small number of studies from low-income countries. Evidence for the effects of vitamin D deficiency on neurobehavioural outcomes from studies in high-income countries might not be generalizable to low-income settings, where impaired child development may be more common, and confounding effects might also differ between these settings. Additionally, isolating the effect of vitamin D on neurobehavioural outcomes may be limited by confounding factors. We excluded two randomized controlled trials that did not evaluate the effect of vitamin D separately from other micronutrients ^77, 78^. Moreover, although most of the observational studies adjusted for measured confounders, evidence may still be subject to residual confounding from unmeasured factors, such as dietary intake of other micro and macronutrients.

## Conclusion

Evidence as to whether prenatal or childhood vitamin D status affects neurobehavioural outcomes in children is limited and inconsistent and further investigation is warranted. For instance, standardized cut-offs for assessing vitamin D deficiency, as well as well-validated and standardized methods of assessing neurobehavioural outcomes would be useful to allow comparison among studies globally. Well-powered randomized controlled trials are required to determine whether vitamin D is beneficial for brain development in children and for optimal dosage and timing of vitamin D supplementation.

## Data availability

### Underlying data

All data underlying the results are available as part of the article and no additional source data are required.

### Extended data

Figshare: Effects of vitamin D deficiency on neurobehavioural outcomes in children: a systematic review-supplementary files. https://doi.org/10.6084/m9.figshare.11799063.v8 ^18^.

This project contains the following extended data:

- Extended datafile 1: Search terms.
- Extended datafile 2. Risk of bias in the included randomized controlled trial based on the Cochrane handbook criteria.
- Extended datafile 3. STROBE checklist assessing the quality of observational studies included in the review.

### Reporting guidelines

Figshare: PRISMA checklist for ‘Effects of vitamin D deficiency on neurobehavioural outcomes in children: a systematic review’. https://doi.org/10.6084/m9.figshare.11799063.v8 ^18^.

Data are available under the terms of the Creative Commons Attribution 4.0 International license (CC-BY 4.0).

## Author information

AMM, SHA and AA conceived the idea of the review and developed the protocol. AMM conducted the literature search, selected studies and extracted relevant information with the guidance of SHA, RMM and AA. AMM, SHA and AA synthesized the data and wrote the first draft of the manuscript. AMM, SHA, AA, RMM, ELW, TNW and AME revised successive drafts of the paper and approved the final version.
