# Supplementary material for: Effects of vitamin D deficiency on neurobehavioural outcomes in children: a systematic review
Source: Wellcome Open Res. 2020 Jun 11;5:28. Originally published 2020 Feb 13. [Version 2] doi: 10.12688/wellcomeopenres.15730.2 (PMC7194460; doi:10.12688/wellcomeopenres.15730.2)
Supplement: Peer review history

# Peer review: Reviewer response for version 2

**DOI:** 10.21956/wellcomeopenres.17601.r39060

**Published:** 2020-06-12

**Authors:** Andrew S. Day

**Affiliations:** 1 Department of Paediatrics, University of Otago, Christchurch, Christchurch, New Zealand

Thank you for revisions

 Manuscript improved with revisions

Are the rationale for, and objectives of, the Systematic Review clearly stated?

Yes

Is the statistical analysis and its interpretation appropriate?

I cannot comment. A qualified statistician is required.

Are sufficient details of the methods and analysis provided to allow replication by others?

Yes

Are the conclusions drawn adequately supported by the results presented in the review?

Yes

Reviewer Expertise:

Paediatric gastroenterology, with focus on IBD and the impact of vitamin D

I confirm that I have read this submission and believe that I have an appropriate level of expertise to confirm that it is of an acceptable scientific standard.

# Peer review: Reviewer response for version 2

**DOI:** 10.21956/wellcomeopenres.17601.r39061

**Published:** 2020-06-11

**Authors:** Mairead Kiely

**Affiliations:** 1 Cork Centre for Vitamin D and Nutrition Research, School of Food and Nutritional Sciences and INFANT Research Centre, University College Cork, Cork, Ireland

Thank you for your comprehensive responses. Approved.

Are the rationale for, and objectives of, the Systematic Review clearly stated?

Yes

Is the statistical analysis and its interpretation appropriate?

Yes

Are sufficient details of the methods and analysis provided to allow replication by others?

Yes

Are the conclusions drawn adequately supported by the results presented in the review?

Yes

Reviewer Expertise:

NA

I confirm that I have read this submission and believe that I have an appropriate level of expertise to confirm that it is of an acceptable scientific standard.

# Peer review: Reviewer response for version 1

**DOI:** 10.21956/wellcomeopenres.17243.r37928

**Published:** 2020-04-21

**Authors:** Mairead Kiely

**Affiliations:** 1 Cork Centre for Vitamin D and Nutrition Research, School of Food and Nutritional Sciences and INFANT Research Centre, University College Cork, Cork, Ireland

This is a comprehensive SLR of vitamin D status and neurological development in children.

 The review includes both trials, of which there were only 2 (both very small), mother-infant prospective cohort studies and cross-sectional studies. Implementation of the search strategy & review are clearly and transparently reported. 

 The results are logically described and well-written. This is a highly heterogenous dataset describing complex outcomes and the authors treated the results objectively.

 A couple of points:

- The Salas paper on preterm infants should not be included as preterm infants are a different and high-risk subgroup from a developmental perspective.
- Although the authors correctly point out the deficits in the trial data, I thought that the discussion could have appraised the evidence basis more from the prospective cohorts from a vitamin D perspective; for example were gold standard methods of 25(OH)D analysis used? Were appropriate methods used for cord blood sampling and analysis?
- Could some sensitivity analysis have been conducted on the basis of 25(OH)D concentrations reported given the widely variant definitions of "vitamin D deficiency"?
- Were the developmental assessments led by clinical psychologists within a MDT? 
- Title: Currently suggestive rather than descriptive - Suggest amendment to: "Inconclusive associations between vitamin D status during pregnancy or at birth and neuro-behavioural outcomes in children: a systematic review."

Are the rationale for, and objectives of, the Systematic Review clearly stated?

Yes

Is the statistical analysis and its interpretation appropriate?

Yes

Are sufficient details of the methods and analysis provided to allow replication by others?

Yes

Are the conclusions drawn adequately supported by the results presented in the review?

Yes

Reviewer Expertise:

Nutrition; Vitamin D; Maternal & Child nutrition and development.

I confirm that I have read this submission and believe that I have an appropriate level of expertise to confirm that it is of an acceptable scientific standard.

# Response

**Published:** 2020-06-03

**Authors:** Sarah Atkinson

**Affiliations:** KEMRI-Wellcome Research Programme, Kenya

We thank the reviewer for their insightful comments. 

**The Salas paper on preterm infants should not be included as preterm infants are a different and high-risk subgroup from a developmental perspective.**

*Response: As noted, extremely preterm neonates are at a higher risk of neurodevelopmental delays compared to children born at full term. We have now excluded the Salas paper as suggested and mentioned it briefly in the Results and Discussion indicating the reason for exclusion as follows:*

*Results under ‘Study Selection’: “We excluded one RCT as it evaluated the effect of vitamin D supplementation on cognitive development in extremely preterm neonates* *[* *1* *]* *.”*

*Discussion under ‘Strengths and limitations’: “We identified another small RCT (n=70) in the USA which reported no beneficial effect of vitamin D supplementation on cognition at two years of age, but we excluded this study as it included extremely preterm neonates, who are at a high risk of neurodevelopmental delays compared to children born at full term* *[* *3* *]* *.”*

*We have updated Figures 2 and 3 to reflect this exclusion.*

**Although the authors correctly point out the deficits in the trial data, I thought that the discussion could have appraised the evidence basis more from the prospective cohorts from a vitamin D perspective; for example, were gold standard methods of 25(OH)D analysis used? Were appropriate methods used for cord blood sampling and analysis?**

*Response: Thank you. We agree that appraisal of the methods used to quantify 25(OH)D levels is important. We have now included the different assays used to quantify serum or plasma 25(OH)D levels in Table 1 and in the Results and Discussion sections as follows:*

*Results under ‘Study characteristics and outcomes’:* “ *The studies used varying definitions for vitamin D status and different assays to quantify 25(OH)D levels (Table 1). All five studies that measured 25(OH)D levels in cord blood did not provide information on the sampling procedures.”*

*Results under ‘Comparing findings based on the timing, assays and cut-offs for 25(OH)D levels and neuroassessment tools.: “The most commonly used assay for measuring 25(OH)D levels was liquid chromatography-tandem mass spectroscopy (LC-MS/MS) (Table 1) with four out of 11 studies that used LC-MS/MS reporting associations between maternal or child 25(OH)D levels and neurobehavioral outcomes, while 13 out of 20 studies that used other assays reported associations* *.* *”*

*Discussion, Paragraph 6: “* *Serum or plasma 25(OH)D levels were quantified using a variety of routinely available assays with most studies using the gold standard of liquid chromatography–tandem mass spectrometry* *(LC-MS/MS). There was little difference in neurobehavioral outcomes in studies that used LC-MS/MS and studies that used other assays to measure 25(OH)D levels.* *The lack of standardized assays may result in large inter-method variability and errors limiting comparability between studies* *[* *2* *]* *.”*

**Could some sensitivity analysis have been conducted on the basis of 25(OH)D concentrations reported given the widely variant definitions of "vitamin D deficiency"?**

*Response: We thank the reviewer for this comment and note that a main limitation of the review is the high level of heterogeneity in the studies which precluded a meta-analysis and formal sensitivity analyses. We have added the following information:*

*Methods under ‘Synthesis of included articles’:* “ *We compared study findings based on when 25(OH)D levels were measured, assays used to measure 25(OH)D levels, definitions of vitamin D status and tools used to assess neurobehavioural outcomes.* *”*

*Results under ‘Comparing findings based on the timing, assays and cut-offs for 25(OH)D levels and neuroassessment tools’: “Six out of eight studies that measured maternal 25(OH)D levels in the 1 ^st^ or 2 ^nd^ trimester reported associations with neurobehavioural outcomes in contrast to three out of eight studies that measured 25(OH)D levels in the 3 ^rd^ trimester or in cord blood. Three out of seven studies that measured 25(OH)D levels in infancy and two out five studies that measured levels in children above one year reported associations with neurobehavioural outcomes (Table 1). The most commonly used assay for measuring 25(OH)D levels was liquid chromatography-tandem mass spectroscopy (LC-MS/MS) (Table 1) with four out of 11 studies that used LC-MS/MS reporting associations between maternal or child 25(OH)D levels and neurobehavioral outcomes, while 13 out of 20 studies that used other assays reported associations. The majority of studies (n=15) defined vitamin D deficiency as 25(OH)D levels <50 nmol/L and six of these studies reported associations with neurobehavioural  outcomes. Six studies defined vitamin D deficiency as 25(OH)D levels <30 nmol/L or <25 nmol/L and of these two reported associations with neurobehavioural outcomes  (Table 1). The most common tool used to assess neurobehavioural outcomes was the Bayley Scales of Infant Development (BSID) and six out of eight studies that used the BSID reported associations between 25(OH)D levels and neurobehavioural outcomes (Table 1).”*

*Discussion under ‘Strengths and limitations’: “We found substantial heterogeneity in study populations and study methods including timing of vitamin D measurements, definitions for vitamin D status and tools used to measure neurobehavioural outcomes which made comparison of the studies difficult and precluded a quantitative meta-analysis of the studies. However, we did not observe marked differences when comparing findings in studies that defined vitamin D deficiency as 25(OH)D levels <50 nmol/L with those that defined vitamin D deficiency as 25(OH)D levels <30 nmol/L or <25 nmol/L.”*

*Discussion Paragraph 6: “Serum or plasma 25(OH)D levels were quantified using a variety of routinely available assays with most studies using the gold standard of liquid chromatography–tandem mass spectrometry (LC-MS/MS). There was little difference in neurobehavioral outcomes in studies that used LC-MS/MS and studies that used other assays to measure 25(OH)D levels. The lack of standardized assays may result in large inter-method variability and errors limiting comparability between studies* *[* *2* *]* *. Secondly, vitamin D status was measured at varying gestational ages in mothers and different ages in the children. Several studies that measured maternal 25(OH)D in the 1 ^st^ or 2 ^nd^  trimester, rather than the 3 ^rd^  trimester* *or in cord blood, reported an association between low maternal 25(OH)D and low developmental scores in the children* ^32^^, ^^41^^, ^^43^^, ^^45^^, ^^46^ *,which might suggest a critical window for the impact of low 25(OH)D levels earlier in pregnancy when there is rapid brain development [71]. There was little difference when comparing findings between studies that measured 25(OH)D levels during or after infancy. In addition, studies suggest that there might be optimal levels of vitamin D required for development with higher levels also being associated with impaired development* *^28^* *^,^* *^40^* *. Thirdly, the timing and tools used to assess neurobehavioural outcomes varied markedly between studies. The majority of studies that assessed neurobehavioural outcomes during infancy reported associations between 25(OH)D levels and neurobehavioral outcomes compared to fewer than half of studies that assessed neurobehavioural outcomes after infancy. Putative effects might be more likely to be detected in earlier years of life and may also be influenced by 25(OH)D levels throughout childhood. Most studies used the Bayley Scales of Infant Development to assess neurobehavioral outcomes in children, however, comparisons based on the tools were limited as most studies used several tools to assess different neurobehavioural outcomes.”*

**Were the developmental assessments led by clinical psychologists within a MDT?**

*Response: We have now included a summary of the teams that assessed neurobehavioural outcomes in the studies in the sub-section ‘Study characteristics and outcomes’ in the Results section as follows:*

*Results under ‘Study characteristics and outcomes’: “In 21 studies, neurobehavioural assessments were conducted by psychologists or trained research or clinical staff, seven studies relied on caregiver reports, while three studies did not provide information on assessors.”*

**Title: Currently suggestive rather than descriptive - Suggest amendment to: "Inconclusive associations between vitamin D status during pregnancy or at birth and neuro-behavioural outcomes in children: a systematic review."** 

*Response: Thank you for the suggestion. However, we note that our review includes one randomised controlled trial and includes studies where vitamin D status was measured in childhood and the suggested title may not be inclusive of all studies.*

# Response

**Published:** 2020-06-11

**Authors:** Mairead Kiely

**Affiliations:** University College Cork, Ireland

Thank you for your comprehensive responses. Approved.

# Peer review: Reviewer response for version 1

**DOI:** 10.21956/wellcomeopenres.17243.r38129

**Published:** 2020-03-23

**Authors:** Andrew S. Day

**Affiliations:** 1 Department of Paediatrics, University of Otago, Christchurch, Christchurch, New Zealand

This MS focuses on the impact of vitamin D upon child development, using a systematic approach.

 Overall comments:

- The MS is well written and easy to follow. The objectives are clear and the conclusions well presented.
- The authors have used we/our throughout rather than third person or indirect language.

Specific comments:

1. The first paragraphs of the Methods and Results sections don't have subheadings - these should be added.
2. The authors used very broad inclusion criteria, for instance including children to age 18 years. A more limited focus, for instance on infancy or the preschool years, may have provided more emphasis on key early aspects of development.
3. Further, vitamin D may be hard to consider as an isolated effect (separate to other micro or macronutrient factors). Was this considered?

Are the rationale for, and objectives of, the Systematic Review clearly stated?

Yes

Is the statistical analysis and its interpretation appropriate?

I cannot comment. A qualified statistician is required.

Are sufficient details of the methods and analysis provided to allow replication by others?

Yes

Are the conclusions drawn adequately supported by the results presented in the review?

Yes

Reviewer Expertise:

Paediatric gastroenterology, with focus on IBD and the impact of vitamin D

I confirm that I have read this submission and believe that I have an appropriate level of expertise to confirm that it is of an acceptable scientific standard.

# Response

**Published:** 2020-06-03

**Authors:** Sarah Atkinson

**Affiliations:** KEMRI-Wellcome Research Programme, Kenya

We thank the reviewer for his helpful comments, which we have addressed point-by-point below:

**The first paragraphs of the Methods and Results sections don't have subheadings - these should be added.** 

*Response: We have added the subheadings ‘Reporting guidelines’ in the Methods section and ‘Study selection’ in the Results section.*

**The authors used very broad inclusion criteria, for instance including children to age 18 years. A more limited focus, for instance on infancy or the preschool years, may have provided more emphasis on key early aspects of development. ** 

*Response: We agree that the early years of life are particularly crucial for brain development. We have included a subsection ‘Studies that assessed neurobehavioural outcomes in infancy’ in the Results section comparing studies that assessed development during infancy and those that assessed development in older children and have also updated the Discussion accordingly as follows:*

*Results, under ‘Studies that assessed neurobehavioural outcomes in infancy’: “Six observational studies assessed neurobehavioural outcomes during infancy and of these studies, five reported associations between maternal or child 25(OH)D levels and neurobehavioural outcomes, while one study reported no associations. In contrast, 24 studies assessed neurobehavioural outcomes in children above one year of age and of these, 11 studies reported associations between maternal or child 25(OH)D levels and neurobehavioural outcomes, while 13 studies reported no associations.”*

*Discussion Paragraph 6: “Thirdly, the timing and tools used to assess neurobehavioural outcomes varied markedly between studies. The majority of studies that assessed neurobehavioural outcomes during infancy reported associations between maternal or child 25(OH)D levels and neurobehavioral outcomes compared to fewer than half of studies that assessed neurobehavioural outcomes after infancy. Putative effects might be more likely to be detected in earlier years of life and may also be influenced by 25(OH)D levels throughout childhood.”*

**Further, vitamin D may be hard to consider as an isolated effect (separate to other micro or macronutrient factors). Was this considered?**

*Response: We agree that the effects of vitamin D on development are possibly confounded by other factors including the effects of other micro and macronutrients. We excluded two RCTs from the systematic review as they did not evaluate the effect of vitamin D on neurobehavioural outcomes in children separately from other micronutrients and have also now discussed this as a potential limitation of the study as follows:*

*Results under ‘Study Selection’: “We excluded 29 studies because the outcomes of interest were physical growth, psychiatric disorders or infections, eight because they assessed coverage of vitamin D supplementation programmes and two RCTs because the effect of vitamin D status on child development was not measured separately from the effects of other micronutrients.”*

*Discussion under ‘Strengths and limitations’: “Additionally, isolating the effect of vitamin D on neurobehavioural outcomes may be limited by confounding factors. We excluded two randomised controlled trials that did not evaluate the effect of vitamin D separately from other micronutrients [4, 5]. Moreover, although most of the observational studies adjusted for measured confounders, evidence may still be subject to residual confounding from unmeasured factors, such as dietary intake of other micro and macronutrients.”*
